# Venous Excess Doppler ultrasound assessment and loop diuretic efficiency in acute cardiorenal syndrome

**DOI:** 10.1186/s12882-025-04060-z

**Published:** 2025-03-27

**Authors:** Eslam Abu-Naeima, Moataz Fatthy, Mahmoud Amin Abu-Sheaishaa Shalaby, Ghada Ayeldeen, Frederik H. Verbrugge, Philippe Rola, William Beaubien-Souligny, Ahmed Fayed

**Affiliations:** 1 Nephrology Unit, Internal Medicine Department, Kasr Al Ainy School of Medicine, Cairo University, Al Kasr Al Ainy, Old Cairo, Cairo Governorate, Cairo, 4240310 Egypt; 2 Cardiology Department, Kasr Al Ainy School of Medicine, Cairo University, Cairo, Egypt; 3 Medical Biochemistry and Molecular Biology Department, Kasr Al Ainy School of Medicine, Cairo University, Cairo, Egypt; 4 Centre for Cardiovascular Diseases, University Hospital Brussels, Jette, Belgium; 5 Faculty of Medicine and Pharmacy, Vrije Universiteit Brussel, Jette, Belgium; 6 Intensive Care Unit, Santa Cabrini Hospital CEMTL, Montreal, Canada; 7 Division of Nephrology, Department of Medicine, Centre Hospitalier Universitaire de Montréal, Montréal, Québec Canada

**Keywords:** Venous congestion, Cardiorenal syndrome, Acute kidney injury, Diuretic efficiency

## Abstract

**Background:**

Cardiorenal syndrome poses significant diagnostic and therapeutic challenges. The Venous Excess Ultrasound (VExUS) grading system based on the combination of venous Doppler assessments has shown potential in predicting acute kidney injury and cardiovascular outcomes, but its relevance regarding the management of acutely decompensated heart failure (ADHF) remains to be fully understood.

**Methods:**

In this prospective study, patients with ADHF and acute kidney injury (AKI) were enrolled from a medical intensive care unit over 20 months. The study involved echocardiography and VExUS grading at admission and 72 h later. Data collection included clinical parameters, diuretic dosages, urine output, and fluid balance. Statistical analyses focused on exploring the relationships between VExUS grades and its components, including the renal venous stasis index (RVSI), diuretic efficiency, and renal function improvement.

**Results:**

The cohort of 43 patients showed varied VExUS grades at admission. Higher VExUS grades were significantly associated with lower diuretic efficiency. Specifically, the mean urine output per 40 mg of furosemide was 368 ± 213 mL, with patients having VExUS grade 2 or 3 exhibiting reduced diuretic efficiency compared to those with grade 0–1 (Grade 2 vs. Grade 0–1: 333 ± 214 mL vs. 507 ± 189 mL, *p* = 0.02; Grade 3 vs. Grade 0–1: 270 ± 167 mL vs. 507 ± 189 mL, *p* = 0.004). The relationship between VExUS grade and diuretic efficiency was independent of admission creatinine and prior use of loop-diuretics (β = -106 CI: -180; -32 *p* = 0.006). Among the components of venous congestion assessment, the RVSI had the best ability to predict low diuretic efficiency (AUROC: 0.76 (0.60; 091) *p* = 0.001). Improvement in VExUS grade at 72 h was correlated with significant renal function improvement (84.6% vs. 47.1% for improved vs. non-improved VExUS grades, *p* = 0.03).

**Conclusion:**

High VExUS and RVSI grades at admission are independently associated with reduced diuretic efficiency in ADHF patients with AKI. The findings emphasize the clinical value of venous congestion assessment in cardiorenal syndrome management including the selection of an initial diuretic dose.

**Supplementary Information:**

The online version contains supplementary material available at 10.1186/s12882-025-04060-z.

## Background

The cardiorenal syndrome remains a challenge to appropriately diagnose, classify, and manage. Traditionally, worsening of kidney function in the setting of acutely decompensated heart failure (ADHF) was felt to be driven by reduced cardiac output. Since then, the recognition of several phenotypes of cardiorenal syndrome has occurred and the importance of venous congestion as the driving hemodynamic mechanism of kidney failure was increasingly recognized [1]. Nonetheless, while pulmonary edema often warrants rapid initial decongestive treatment, it is often unclear how the evaluation of systemic congestion through various modalities should modify the treatment strategy during hospitalisation. Beyond resulting in congestive cardiorenal syndrome, venous congestion of the kidney is also known to contribute to diuretic resistance in some but not all patients [2–4].

Venous Doppler can identify markers of reduced venous compliance at the bedside of patients with ADHF. Recently, a composite score of solid abdominal organ Doppler coined as the Venous Excess Ultrasound (VExUS) assessment showed a strong association with the development of acute kidney injury (AKI) in a cohort of Cardiac surgery patients [5], while alterations in intra-renal venous Doppler were demonstrated to predict cardiovascular mortality or repeat hospitalizations in patients with heart failure [6] or pulmonary hypertension [7]. Two observational study of patients with cardiorenal syndrome showed an association between the reduction of venous Doppler markers and improvement in renal dysfunction [8, 9]. However, since most of these patients with ADHF would have received decongestive treatment on the basis of other clinical findings, it is unclear how the additional information from VExUS impacted management. In this regard, in the context of increasing use of VExUS in routine care is an opportunity to gain further insights about the clinical significance of these markers beyond their prognostic significance.

Physiologically, the importance of systemic venous congestion has been recognized for almost a century [10] although the concepts of fluid tolerance and congestive organ dysfunction as clinical priorities are fairly modern. Over a decade ago, Goonewardena et al. showed that a single measure of the IVC - if over 20 mm - at discharge was sufficient to predict a higher risk of readmission [11]. Today, the democratisation of high-quality ultrasound devices has now given clinicians powerful tools to use at the bedside to complement other components of the clinical evaluation providing a panel of physiologic targets that could be used to guide treatment and not only prevent readmission but also improve organ function.

In this study, we hypothesised that a higher VExUS grade at admission might be associated with reduced diuretic efficacy in patients with ADHF. We also aimed to report the prevalence and evolution of VExUS during the first 72 h of hospital stay as well as the association with the change in renal function during this period.

## Methods

### Setting

This is a prospective study that recruited patients with a provisional diagnosis of cardiorenal syndrome admitted in the medical intensive care unit in Kasr Al Ainy Hospital, Cairo University during a period of 20 months from September 2021 to April 2023. Recruitment was only active when trained operators were available. Written consent was obtained for all patients and the study protocol was approved by Cairo university faculty of medicine Research Ethics Committee (MD-312-2021). The reporting is done in accordance with the Strengthening the Reporting of Observational Studies in Epidemiology (STROBE) Statement [12].

### Participants

We enrolled patients over 18 years of age who presented with a clinical diagnosis of ADHF and AKI at admission. The eligibility was assessed by a cardiologist and a nephrologist. ADHF was defined as a diagnosis of heart failure with at least one clinical sign of volume overload (edema, effusions, dyspnea). AKI was defined according to kidney disease improving global outcomes (KDIGO) 2012 Clinical practice guidelines for Acute Kidney Injury [13], it was diagnosed by an absolute increase in sCr, at least 0.3 mg/dL (26.5 µmol/L) within 48 h, by a 50% increase in sCr from baseline, or by a urine output of less than 0.5 mL/kg/h for at least 6 h. Baseline creatinine was based on outpatient measurements available in the last 3 months or when unavailable, a back formula was used to calculate the baseline creatinine. Serum creatinine = (75/186 × (age^− 0.203^) × (0.742 if female) × (1.21 if black)])^− 0.887^ [14]. We excluded patients with a diagnosis of obstructive AKI, those having received contrast exposure, known for stage 5 chronic kidney disease or liver cirrhosis, the critically ill at admission (receiving either vasopressor medication or mechanical ventilation, and patients without adequate ultrasound windows, or with inferior vena cava (IVC) thrombosis.

### Ultrasound assessment

Patients underwent ultrasound evaluation by researchers who were also the treating physicians of the participants on admission and 72 h later. A phased array probe was used.

The evaluation included lung ultrasound, IVC, and pulse wave venous Doppler of the hepatic, portal and intra-renal veins as previously described [15]. The subxiphoid or lateral transhepatic windows (when the subxiphoid window was not appropriate) were used to assess the hepatic vein and the portal vein. A simultaneous electrocardiographic (ECG) tracing was used to identify the components of the hepatic venous waveforms. The IRVD was performed on the inter-lobar vessels which were scanned in the lateral mid-axillary window [16]. The VExUS grade was derived as proposed by Beaubien-Souligny W, et al. as in figure S1 [5].

The VExUS grading system was interpreted as follows:

- Grade 0: IVC < 20 mm.
- Grade 1: IVC ≥ 20 mm with normal patterns or mild abnormalities.
- Grade 2: IVC ≥ 20 mm with severe abnormality in at least one pattern.
- Grade 3: IVC ≥ 20 mm with severe abnormalities in multiple patterns.

The renal venous stasis index (RVSI) was calculated as follows: (cardiac cycle time – venous flow time)/ cardiac cycle time [7]. Also, echocardiographic parameters were recorded on admission including, left and right ventricles diameters, filling pressures, left ventricle ejection fraction (LVEF), tricuspid annular plane systolic excursion (TAPSE), Pulmonary artery systolic pressure (PASP), and right ventricular fractional area of change (RV FAC).

### Data collection

Data collection from the participant file included: clinical parameters including body weight, invasive central venous pressure monitoring (CVP), and sequential organ failure assessment (SOFA) score [17], N-terminal pro-beta natriuretic peptide (NT-proBNP), sodium and chloride profiles.

All our patients received furosemide, which was given as 2–3 daily boluses or as a continuous infusion. All the patients were managed according to ESC Guidelines for the Diagnosis and treatment of acute and chronic heart failure [18]. All diuretic doses, urine output (UOP), fluid balance, change in body weight (BW), and diuretic efficiency were calculated and documented. Loop diuretic efficiency was assessed as urine output and change in body weight. We evaluated the cumulative urine output in milliliters (mL) divided by the total furosemide dose in milligrams. expressed as mL per 40 mg equivalent of furosemide as previously proposed [19]. We followed serum creatinine level on discharge. Also, mortality and re-hospitalization during the first 90 days of discharge by phone calls.

### Statistical analysis

Categorical data is presented in N (%) while continuous data is presented in mean ± standard deviation or median (interquartile range) based on the distribution of data on Q-Q plot to assess the normal distribution. Patients’ characteristics were compared according to categories of VExUS 0–1, 2 and 3 using Chi-squared or Fisher’s exact test for categorical variables, or one-factor ANOVA or Kurskall-Wallis test for continuous variable depending of whether a normal distribution is present. When significant results *p* < 0.05 are found, post-hoc pairwise comparisons with Bonferonni correction were performed and any significant association are reported in the legends of tables.

To assess the association between diuretic efficiency and VExUS, a multivariable linear regression model was used with the following adjustment variables: admission creatinine and prior use of loop-diuretics at home. These variables were selected a-priori as factors that are commonly associated with lesser loop-diuretics efficiency. Results are presented as non-standardized regression coefficients. The correlation between RVSI and diuretic efficiency was also assessed using spearman correlation coefficient.

As sensitivity analyses, we categorized patients between low (< 325mL) and high diuretic efficiency (≥ 325 mL) based on the median cumulative urine output per 40 mg of furosemide equivalent. We evaluated the ability of VExUS grading and other markers to discriminate between low and high diuretic efficiency using Receiver operating characteristic analysis (ROC). The results are presented as plots and areas under the ROC curve (AUROC) with 95% confidence intervals. We also constructed exploratory multivariable logistic regression models to assess the ability of the VExUS grade to predict a low diuretic efficiency after adjustments with other potential clinical markers. The results are presented as odds ratio (OR) with 95% confidence intervals.

All analyses were performed in SPSS version 29.0 and considered *p* < 0.005 as significant.

## Results

### Patient characteristics

The cohort was composed of 43 patients of whom 12 (27.9%) had a VExUS grade of 3, 18 (41.9%) had a VExUS grade of 2, and 13 (30.2%) had a VExUS grade 0 or 1. Patients’ characteristics are presented in Table 1. The cohort was composed in equal proportion of patients with preserved and reduced left ventricle ejection fraction (LVEF). The mean age was 52 ± 15 years with an important proportion of patients having known arterial hypertension, diabetes, and prior coronary artery disease. Participants had signs of congestion on physical examination including a high prevalence of lung rales and lower extremity edema. The initial serum creatinine was 2.59 ± 1.58 mg/dL with a substantial proportion of patients with criteria compatible with severe AKI (stage 2: 20.9%, Stage 3: 16.3%).

**Table 1:** Patients characteristics at admission and outcomes in relationship with the initial vexus grading

Characteristics	All patients *N* = 43	VExUS Grade 0–1 (*N* = 13)	VExUS Grade 2 (*N* = 18)	VExUS Grade 3 (*N* = 12)	*p*-value
Baseline characteristics					
Age	52 ± 15	54 ± 16	51 ± 14	52 ± 17	0.92
Male sex	25 (56.8%)	7 (53.8%)	13 (72.2%)	5 (41.7%)	0.23
Chronic hypertension	30 (69.8%)	8 (61.5%)	18 (72.2%)	9/12 (75.0%)	0.73
Diabetes	23 (53.5%)	4 (30.8%)	12 (66.7%)	7 (58.3%)	0.13
Known chronic kidney disease	6 (14%)	1 (7.7%)	3 (16.7%)	2 (16.7%)	0.74
Chronic obstructive pulmonary disease	11 (25.6%)	3 (23.1%)	6 (33.3%)	11 (25.6%)	0.68
Type of heart failure - Preserved LVEF - Reduced LVEF (≤ 40%)	22 (51.2%) 21 (48.8%)	7 (53.8%) 6 (46.2%)	9 (50.0%) 9 (50.0%)	6 (50.0%) 6 (50.0%)	1
Prior hospitalization for acute heart failure	14 (32.6%)	3 (23.1%)	8 (44.4%)	3 (25.0%)	0.39
Coronary artery disease	20 (46.5%)	5 (38.5%)	11 (61.1%)	4 (46.5%)	0.29
Heart rate	98 ± 13	99 ± 13	96 ± 11	100 ± 15	0.64
Systolic blood pressure	134 ± 27	133 ± 28	135 ± 26	134 ± 29	0.98
Diastolic blood pressure	80 ± 14	79 ± 16	81 ± 14	80 ± 14	0.94
Mean arterial pressure	98 ± 18	97 ± 20	99 ± 18	98 ± 18	0.97
Central venous pressure	20 ± 3	19 ± 3	20 ± 3	21 ± 3	0.25
Serum creatinine at admission	2.59 ± 1.58	2.01 ± 0.94	3.10 ± 2.11	2.45 ± 0.90	0.16
NT-pro-BNP	1151 ± 343	1079 ± 314	1116 ± 288	1283 ± 431	0.29
AKI staging at admission - Stage 1 - Stage 2 - Stage 3	27 (62.8%) 9 (20.9%) 7 (16.3%)	11 (84.6%) 1 (7.7%) 1 (7.7%)	11 (61.1%) 2 (11.1%) 5 (27.8%)	5 (41.7%) 6 (50.0%) 1 (8.3%)	0.16
Other congestion parameters					
Serum electrolytes - Sodium (mmol/L) - Chloride (mmol/L)	135 (± 5) 102 (± 14)	134 (± 4) 106 (± 9)	135 (± 4) 106 (± 11)	134 (± 6) 93 (± 18)	0.66 0.03
Pulmonary rale on auscultation	36 (83.7%)	10 (76.9%)	14 (76.9%)	12 (77.8%)	0.25
Lower extremity edema	35 (81.3%)	7 (53.8%)	18 (100%)	10 (83.3%)	0.005
Pleural effusion	28 (58.1%)	6 (46.2%)	10 (55.6%)	9 (75.0%)	0.36
Abdominal Ascites	6 (14.0%)	0 (0%)	0 (0%)	6 (50.0%)	< 0.001
New York Heart Association functional class - 1 - 2 - 3 - 4	0 (0%) 0 (0%) 14 (32.6%) 29 (67.4%)	0 (0%) 0 (0%) 7 (53.8%) 6 (46.2%)	0 (0%) 0 (0%) 6 (33.3%) 12 (66.7%)	0 (0%) 0 (0%) 1 (8.3%) 11 (91.7%)	0.048
Outcomes					
Improvement in renal function at 72 h	31 (72.1%)	13 (92.3%)	18 (55.6%)	12 (75.0%)	0.08
Initiation of RRT during hospital stay	8 (18.6%)	1 (7.7%)	7 (38.9%)	3 (25.0%)	0.15
Death during hospital stay	11 (25.6%)	2 (15.4%)	3 (16.7%)	12 (25.0%)	0.79
3 month re-admission in survivors	7 (20.0%)	1 (11.1%)	5 (41.7%)	1 (16.7%)	0.31

Comparisons were performed using the chi square test or Fisher exact test where appropriate. Post-hoc multiple comparisons were performed with Fisher’s LSD correction: *significant for Grade 1 vs. 3. ^&^significant for Grade 1 vs. 3 and grade 2 vs. 3. LVEF: Left ventricular ejection fraction, RRT: Renal replacement therapy

Baseline characteristics in terms of prior health history did not differ significantly in relationship with the initial VExUS grade. Participants with an elevated VExUS grade were more likely to have lower extremity edema and the presence of abdominal ascites was only seen in patients with an initial VExUS grade of 3. In terms of echocardiographic parameters, patients with high VExUS grade had a higher maximal IVC diameter but the presence of pulmonary B-lines did not differ between groups (Table 2). No significant differences were observed in echocardiographic parameters of left ventricle (LV) and right ventricle (RV) function, as well as with the estimation of pulmonary artery systolic pressure (PASP).

**Table 2:** Ultrasound parameters in relationship with the initial vexus grading

Characteristics	All patients *N* = 43	VExUS Grade 0–1 (*N* = 13)	VExUS Grade 2 (*N* = 18)	VExUS Grade 3 (*N* = 12)	*p*-value
Extra-cardiac ultrasound parameters					
IVC maximal diameter	2.5 ± 0.4	2.3 ± 2	2.5 ± 0.2	2.8 ± 0.5	0.005*
IVC colllapsibility	15.0 ± 9.2	20.1 ± 11.1%	13.2 ± 7.9%	12.3 ± 7.2%	0.054
Hepatic Doppler S/D ratio	0.56 ± 0.49	0.82 ± 0.36	0.71 ± 0.47	0.06 ± 0.2	< 0.001^&^
Portal Doppler pulsatility	52.8 ± 28.5	37.8 ± 5.7%	47.9 ± 8.9%	79.4 ± 45%	< 0.001^&^
Renal venous stasis index	0.36 ± 0.31	0.17 ± 0.20	0.32 ± 0.29	0.63 ± 0.24	< 0.001^&^
B-lines on lung ultrasound	31 ± 10	31 ± 10	30 ± 12	32 ± 8	0.89
Echocardiography parameters					
LVEF (%)	40 ± 14	41 ± 11	40 ± 16	38 ± 15	0.87
Mitral E/e`	15.2 ± 4.8	15.4 ± 4.9	15.4 ± 4.8	14.6 ± 5.0	0.88
PASP	57 ± 11	56 ± 12	57 ± 10	56 ± 12	0.94
TAPSE (mm)	17.4 ± 4.6	15.9 ± 3.8	19.4 ± 5.4	15.9 ± 3.7	0.05
RV FAC (%)	33 ± 14	28 ± 12	36 ± 14	33 ± 16	0.33

Legend: IVC: Inferior vena cava, LVEF: Left ventricular ejection fraction, TAPSE: Tricuspid annular plane systolic excursion, RV FAC: Right ventricular fractional area of change

At follow-up, 11 (25.6%) patients died during hospital stay. In terms of outcomes when considering the initial VExUS grading at admission, no statistically significant differences were found in terms of improvement of kidney function, in-hospital mortality, or re-hospitalisation at 3 months (Table 1).

### Diuretic responsiveness

Loop diuretic use, urine output and cumulative fluid balance in the 72 h follow-up period are presented in Table 3. The average daily dose as well as the cumulative dose of loop diuretics during the observation period was higher in patients with a high VExUS grade with the resulting cumulative fluid balance being similar.

**Table 3:** Loop diuretics and fluid balance in the 72-hour period after admission

	VExUS 0–1	VExUS 2	VExUS 3	*p*-value
Furosemide use - Cumulative dose (mg) - Average dose/day (mg)	480 (360; 720) 160 (120; 240)	720 (480; 1440) 240 (160; 480)	1260 (720; 1440) 420 (240; 480)	0.006^1^ 0.006^2^
Fluid balance - Cumulative (L) - Average per day (L) - Change in body weight (Kg) - Cumulative urine output (L)	-3.2 (-2.1; -3.6) -1.1 (-0.7; -1.2) -1.5 (-1.0; -2.0) 6.3 (5.7; 6.9)	-2.1 (-1.8; -4.6) -0.7 (-0.6; -1.5) -1.5 (-0.0; -3.5) 5.8 (4.6; 7.9)	-4.3 (-2.3; -5.4) -1.4 (-0.8; -1.8) -2.0 (-1.3; -2.8) 6.8 (5.1; 8.3)	0.50 0.40 0.52 0.62

Legend: Mann U witney test, Pairwise comparision with Bonferonni correction: ^1–2^. Significant difference between VExUS grade 1 vs. 3 (*p* = 0.005)

**Fig. 1:**
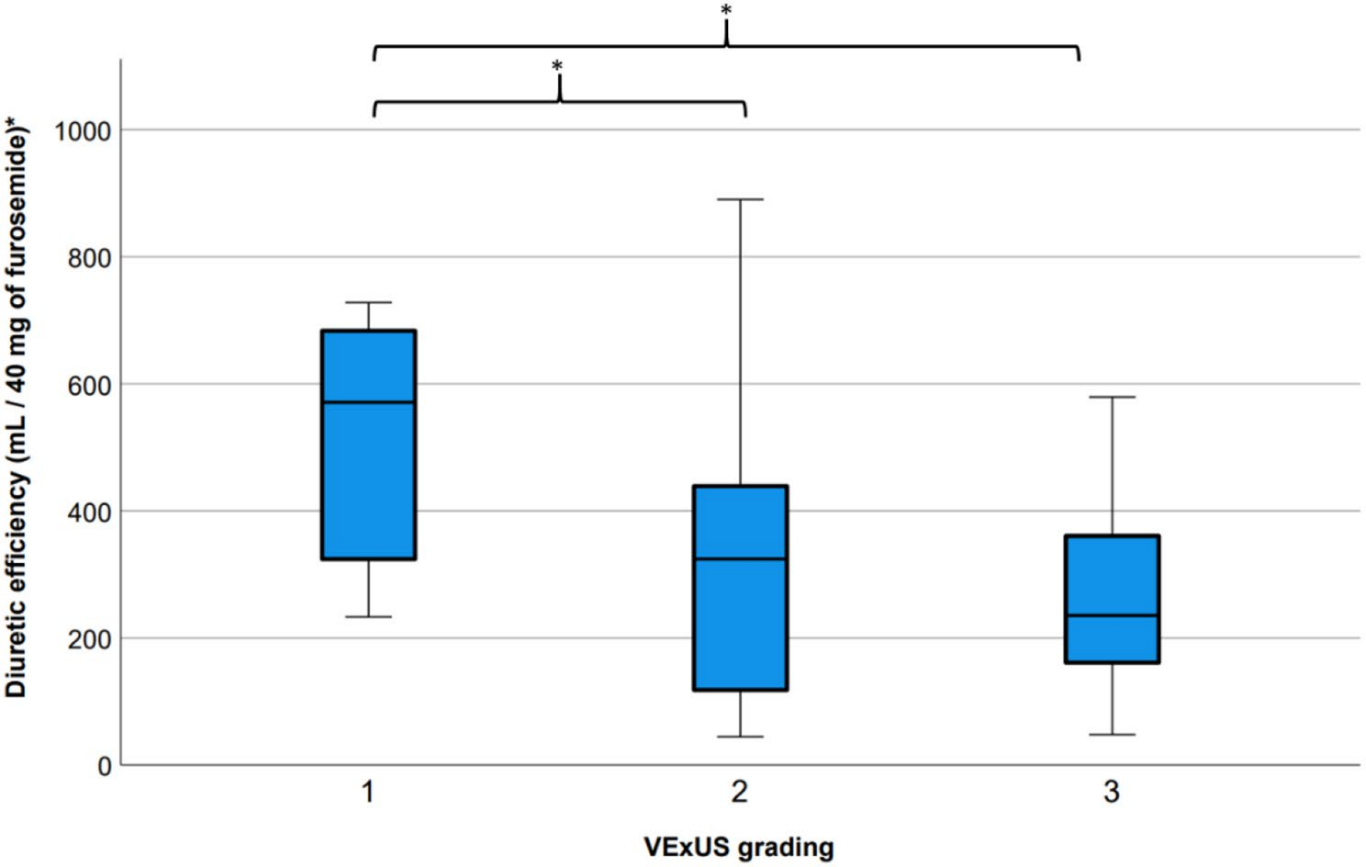
Diuretic efficiency in relationship with VExUS grading and renal venous stasis index at admission. Legend: One-way ANOVA reveal a significant difference in the distribution of the 3 groups (p = 0.01). Post-hoc pair-wise comparisons reveals Fischer’s LSD correction show significant differences between the distribution of VExUS grading 1 vs. 2 (p = 0.02) and group 1 vs. 3 (p = 0.004)

When considering the total urine output and the cumulative loop-diuretic dose administered, the mean urine output per 40 mg of furosemide was 368 ± 213 mL/40 mg. Patients with VExUS grade 2 or grade 3 appeared to have reduced diuretic efficiency compared to patients with grade 0–1 at baseline (Grade 2 vs. Grade 0–1: 333 ± 214 vs. 507 ± 189 mL/40 mg, *p* = 0.02; Grade 3 vs. grade 1: 270 ± 167 vs. 507 ± 189 mL/40 mg, *p* = 0.004) as shown in Fig. 1. Furthermore, a moderate correlation (r=-,377 *p* = 0.01) was observed between the renal vein stasis index (RVSI) and diuretic efficiency (Figure S2).

After adjustment of admission creatinine s well as having received loop diuretics prior to hospital admission, a significant association was found between the initial VExUS grade and diuretic efficiency (β = -106 CI: -180; -32 *p* = 0.006, per each 1 point increase in VExUS grading - Supplementary Table S3). A similar association was also found between RVSI and diuretic efficiency (β = -28.3 CI: -47.0; -9.6 *p* = 0.004, per each 0.1 point increase in RVSI - Supplementary Table S4).

In sensitivity analyses, we found that VExUS grading had a moderate ability to identify patients with a low diuretic efficiency (AUROC: 0.71 (0.56; 0.87) *p* = 0.007) (Table S2). The renal venous stasis index had the best discriminative capability (AUROC: 0.76 (0.60; 091) *p* = 0.001) (Table S2 and Figure S3). In additional analyses, VExUS grade 3 remained independently associated with low diuretic efficiency with adjustment for baseline creatinine, NT-pro-BNP use of loop-diuretic at home, IVC maximal diameter, and CVP (Table S5 of the supplementary material).

### Change in vexus at 72 h and associated factors

Among patients with VExUS grade 2 or 3 at admission (*n* = 30), 13 (43.3%) patients had a reduction of VExUS grading to 0–1 at 72 h while no significant improvement was seen in 17 (56.7%) patients. Patients with an improvement in VExUS had a greater diuretic efficiency as well as a larger decrease in weight, CVP and NT-pro-BNP (Table S1). Furthermore, patients with improvement of the VExUS grade were more likely to have a reduction of serum creatinine at 72 h compared with baseline (84.6% vs. 47.1%, *p* = 0.03). Compared with CVP, a greater relative decrease was observed for portal pulsatility fraction (-34.6% vs. -9.5%, *p* < 0.001) and renal venous stasis index (-36.3% vs. -9.5%, *p* < 0.001) (Figure S4 of the supplementary material).

## Discussion

In a cohort of patients admitted with ADHF and reduced kidney function, we report an independent association between VExUS grade/RVSI and diuretic responsiveness. Additionally, we demonstrate a strong link between significant improvement in venous congestion and improvement of renal function, nearly twice as much as in those for whom decongestion was not achieved.

Diuretic resistance is a critical issue when managing patients with cardiorenal syndrome in the setting of ADHF and is correlated with the prognosis of heart failure in general [19, 20]. It can be due to multiple factors including the severity of kidney impairment -generally taken into account - to guide the initial loop-diuretic dosage [21]. Since rapid and effective decongestive treatment is associated with improved outcomes [22], a prompt escalation of diuretic treatment in cases of sub-optimal initial response based on urine output or spot urine sodium has been advocated [23]. The results of the present study suggest that the severity of venous congestion as determined through the identification of venous Doppler markers could also be clinically relevant as it is independently associated with a reduced diuretic efficiency. Previously, Nijst et al. showed in an experimental setting that heart failure outpatients with alterations in intra-renal venous Doppler in response to fluid expansion had a reduced diuretic response compared with patients with a normal assessment [24]. We thereby confirm this observation for the first time in an acute care setting. Furthermore, our data suggest that the RVSI is the most predictive of low diuretic efficiency. Interestingly, despite a reduced diuretic efficiency, this did not seem to affect the ability to obtain a similar negative fluid balance during the first 72 h. Since the information from VExUS was integrated in clinical care in this cohort, there is a possibility that the diuretic dose was escalated partially in response to these markers although this cannot be formally demonstrated in study.

While VExUS score at admission is not predictive of kidney function improvement, we confirmed the observation reported by two previous reports [8, 9] that kidney function tends to improve in patients in whom VExUS grade or RVSI decrease after admission. Patients who did not show improvement of VExUS grade in the 72 h following admission may represent a subgroup of patients with a higher illness severity and predominance of right-sided heart disease, particularly in the patients with severe pulmonary hypertension and right ventricular limitation or dysfunction, for whom decongestive treatment despite being effective may be not sufficient to restore normal venous compliance and for whom a certain degree of residual congestion is to be expected, or else that decongestion may at some point begin to decrease forward flow and negate the potential improvement in congestion in terms of renal perfusion. Alternatively, the persistence of abnormal venous Doppler may also identify patients for whom therapy should be significantly escalated through either combination diuretic therapy or mechanical fluid removal (ultrafiltration). Decongestive treatment requires significant individualization, and in real-world circumstances, ins and outs, patient weight, jugular venous pressure assessment and even patient symptomatology may not be precise enough to decide when to settle on a dose or escalate further.

This study’s strength is the novelty of investigating venous Doppler markers in a clinical care setting with a particular focus on initial treatment response. The limitations include the small sample size that limits the ability to identify subgroups, perform multivariable adjustment, as well as limiting the power to detect an association with relevant patient-outcomes beyond short-term changes in kidney function. Also, the sample size is quite small which reduce our ability to study the relationship between ultrasound markers and patients’ outcomes. Some data was not collected including precise baseline diuretic dose before admission and urine sodium during furosemide treatment. Inter and intra-observer variability testing were not performed although previous studies on VExUS and its component reported good reproducibility [15, 25, 26]. Finally, the attending physicians were aware of the results of the ultrasound assessment which could have led to modification in their management. However, the primary outcome of diuretic efficiency is unlikely to have been affected.

## Conclusion

This study puts yet another stone in the foundation supporting the need to assess the severity of venous congestion and bringing forward the novel potential of identifying diuretic resistance in patients with cardio-renal issues in order to optimize pharmacologic and/or extracorporeal fluid management. From a clinical standpoint, the presence of an elevated VExUS score or low RVSI should alert the clinician to the likelihood of diuretic resistance, and prompt a close follow-up in order to optimize decongestion. Clinicians need to be aware that congestion can be a significant factor in the management of AKI which should be taken into consideration in randomised trials in order to avoid being covertly undermined by patient heterogeneity in terms of venous congestion, which is rarely assessed precisely. We hope that others can use this study as a springboard to the next levels of interventional research to continue to further the science of precision medicine and fine tune the goals of decongestion, as much remains to be discovered.

## Electronic supplementary material

Below is the link to the electronic supplementary material.

Supplementary Material 1

## Data Availability

Data is provided within the manuscript or supplementary information files.

## Abbreviations

ADHF: Acutely decompensated heart failure
AKI: Acute kidney injury
CVP: Central venous pressure
NT-pro-BNP: N-terminal pro-beta natriuretic peptide
SOFA: Sequential organ failure assessment
VExUS: Venous excess ultrasound
KDIGO: Kidney disease improving global outcomes
ICU: Intensive care unit
2D: Two dimensional
BW: Body weight
UOP: Urine output
mL: milliliter
LV: Left ventricle
RV: Right ventricle
LVEF: Left ventricle ejection fraction
PASP: Pulmonary artery systolic pressure
MPI: Myocardial performance index (MPI)
RV FAC: Right ventricular fractional area of change
RVSI: Renal vein stasis index
