# Supplementary material for: Venous Excess Doppler ultrasound assessment and loop diuretic efficiency in acute cardiorenal syndrome
Source: BMC Nephrol. 2025 Mar 27;26:157. doi: 10.1186/s12882-025-04060-z (PMC11951500; doi:10.1186/s12882-025-04060-z)

**Venous Excess Doppler Ultrasound Assessment and Loop Diuretic Efficiency in**

**Acute Cardiorenal Syndrome**

**Supplementary material:**

Supplementary tables:

Table S1: Characteristics of participants with a high VExUS (Grade 2 or 3) at admission in relationship with the presence of improvement at day 3 (N=30).

|  | **No improvement: VExUS 2-3 at day 3**  **(17/30)** | **Improvement:**  **VExUS 0-1 at day 3**  **(13/30)** | **p-value** |
| --- | --- | --- | --- |
| **Diuretic efficiency*** | 244 ±168 | 391 ±205 | 0.04 |
| **Average daily IV furosemide (mg)** | 468 ±303 | 329 ±232 | 0.18 |
| **Cumulative fluid balance (L)** | 2.9 ±2.0 | 3.9 ±2.0 | 0.16 |
| **Change in weight (Kg)** | 1.1 ±1.5 | 2.5 ±1.3 | 0.005 |
| **Change in CVP (cm H2O)** | -1 ±2 | - 3 ±2 | 0.02 |
| **Change in serum sodium (mmol/L)** | -1 ±4 | 0 ±7 | 0.79 |
| **Change in serum chloride (mmol/L)** | 3 ±13 | -7 ±13 | 0.06 |
| **Change in NT-pro-BNP (umol/L(** | -151 (-640; -16) | -729 (-850; -598) | 0.04 |
| **AKI improvement at 72 hours** | 8 (47.1%) | 11 (84.6%) | 0.03 |
| **In-hospital mortality** | 4 (23.5%) | 2 (15.4%) | 0.67 |

Legend: AKI: Acute Kidney injury, CVP: Central venous pressure.

Table S2: Area under the receiver operating characteristic curve (AUROC) for VExUS grading and other parameters to identify patients with high vs low diuretic efficiency.

| **Marker** | **AUROC (CI) p-value** |
| --- | --- |
| **VExUS grading** | 0.72 (0.56; 0.87) p=0.007 |
| **Renal venous stasis index** | 0.76 (0.60; 0.91) p=0.001 |
| **Hepatic vein S/D ratio** | 0.63 (0.46; 0.81) p=0.14 |
| **Portal vein pulsatility** | 0.59 (0.42; 0.77) p=0.29 |
| **IVC maximal diameter** | 0.61 (0.44-0.78) p=0.21 |
| **NT-pro-BNP** | 0.59 (0.41; 0.76) p=0.33 |

Table S3: Association between the VExUS classification and diuretic efficiency

|  | **Model 1** | | **Model 2** | |
| --- | --- | --- | --- | --- |
|  | **Estimate (CI)** | **p-value** | **Estimate (CI)** | **p-value** |
| **VExUS grading (per 1 point increase)** | -106 (-180; -32) | 0.006 | -98.4 (-175,2; -21.7) | 0.01 |
| **Creatinine at day 1 (for each increase in 1 mg/dL)** | -52 (-90; -15) | 0.008 | -51.3 (-89.2; -13.4) | 0.009 |
| **Use of loop diuretic at home** | 36 (-89; 161) | 0.56 | 49.1 (-80.4; 178.6) | 0.45 |
| **Central venous pressure** |  |  | -7.8 (-27.5; 11.8) | 0.43 |

Legend: Multivariable linear regression for the association with mean urine output per 40 mg of furosemide in the first 3 days.

Table S4: Association between the renal venous stasis index and diuretic efficiency

|  | **Model 1** | | **Model 2** | |
| --- | --- | --- | --- | --- |
|  | **Estimate (CI)** | **p-value** | **Estimate (CI)** | **p-value** |
| **Renal venous stasis index (per 0.1 increase)** | -28.3 (-47.0; -9.6) | 0.004 | -27.8 (-46.3; -93.1) | 0.004 |
| **Creatinine at admission (for each increase in 1 mg/dL)** | -59 (-96; -22) | 0.002 | -56.7 (-93.3; -20.1) | 0.003 |
| **Use of loop diuretic at home** | 12 (-113; 138) | 0.84 | 32.9 ( -94.6; 160.4) | 0.61 |
| **Central venous pressure** |  |  | -12.7 (-31.3; 5.9) | 0.17 |

Legend: Multivariable linear regression for the association with mean urine output per 40 mg of furosemide in the first 3 days.

Table S5: Association between Venous Excess Ultrasound (VExUS) grading and low diuretic efficiency

|  | **Univariable**  **OR (CI) p-value** | **Model 1**  **OR (CI) p-value** | **Model 2**  **OR (CI) p-value** | **Model 3**  **OR (CI) p-value** |
| --- | --- | --- | --- | --- |
| **VExUS**  - **Grade 0-1**  - **Grade 2**  - **Grade 3** | Ref  3.33 (0.68; 16.30) p=0.14  10.00 (1.59; 62.73) p=0.01 | Ref  2.62 (0.438; 15.65) p=0.29  19.29 (1.78; 208.98) p=0.02 | Ref  4.11 (0.79; 21.46) p=0.09  17.14 (1.89; 155.49) p=0.01 | Ref  2.66 (0.44; 16.01) p=0.29  17.33 (1.57; 191) p=0.02 |
| **Creatinine at day 1** | 1.87 (1.02; 3.42) p=0.04 | 1.97 (1.08; 3.59) p=0.03 |  | 1.94 (1.06; 3.54) p=0.03 |
| **NT-pro-BNP at day 1** | 1.03 (0.86; 1.23) p=0.74 | 0.91 (0.71; 1.17) p=0.46 |  | 0.47 (0.71; 1.17) p=0.47 |
| **Use of home loop diuretics** | 1.07 (0.30; 3.84) p=0.92 | 0.69 (0.12; 3.92) p=0.68 |  | 0.60 (0.10; 3.76) p=0.60 |
| **IVC maximal diameter** | 1.46 (0.27; 8.07) p=0.66 | 0.30 (0.03; 3.26) p=0.32 | 0.35 (0.04; 2.85) p=0.33 | 0.31 (0.02; 3.26) p=0.64 |
| **CVP at baseline** |  |  |  | 1.06 (0.82; 1.38) p=0.64 |

Legend: Low diuretic efficiency was defined as less than 325 mL of urine per 40 mg of furosemide equivalent. CI: Confidence interval, OR: odds ratio, IVC: Inferior vena cava

Supplementary figures:

Figure S1: Ultrasound findings by location with progressive abnormality; and the VExUS grading system interpretation.


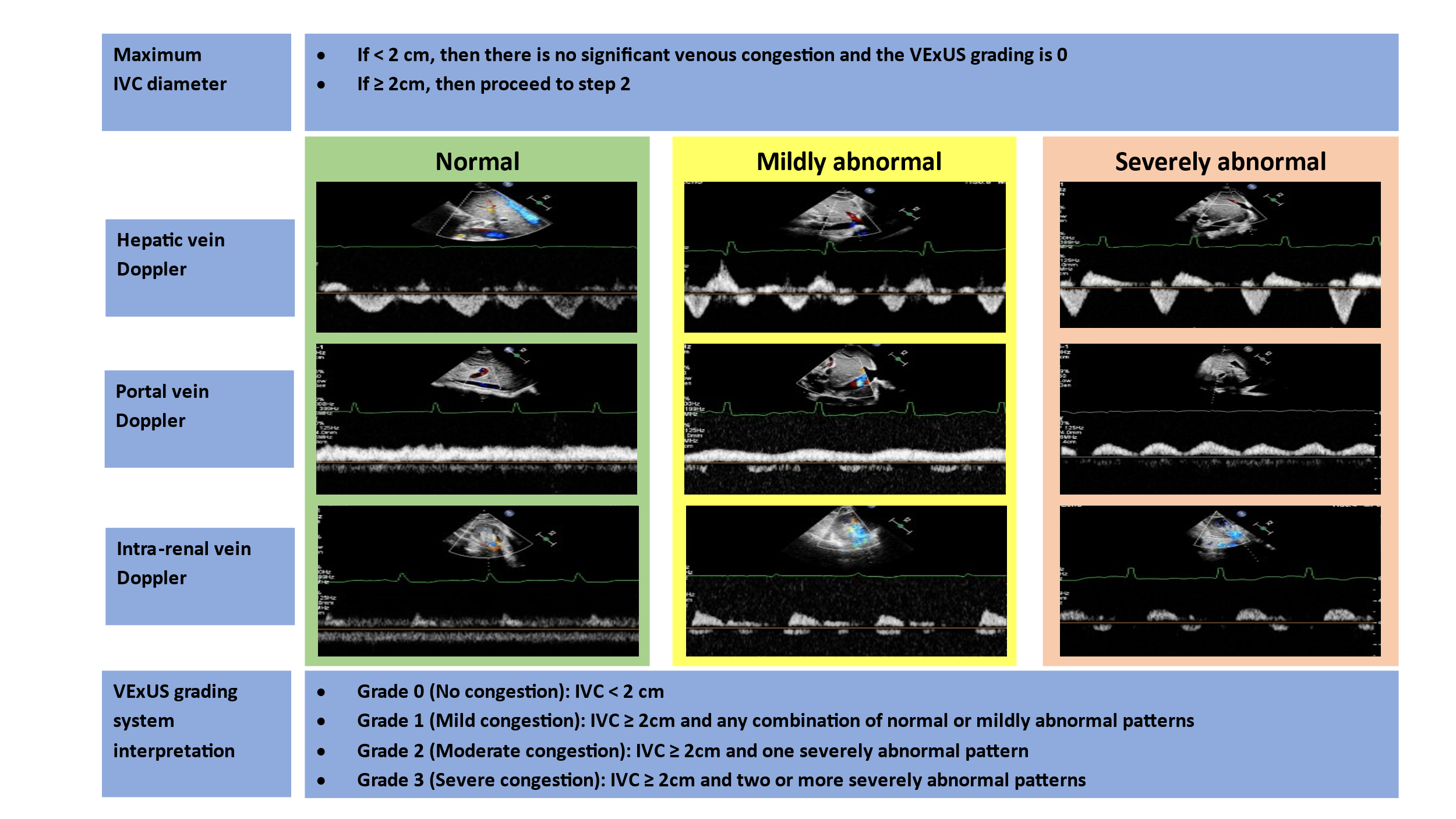


Figure S2: Diuretic efficiency in relationship with renal venous stasis index at admission


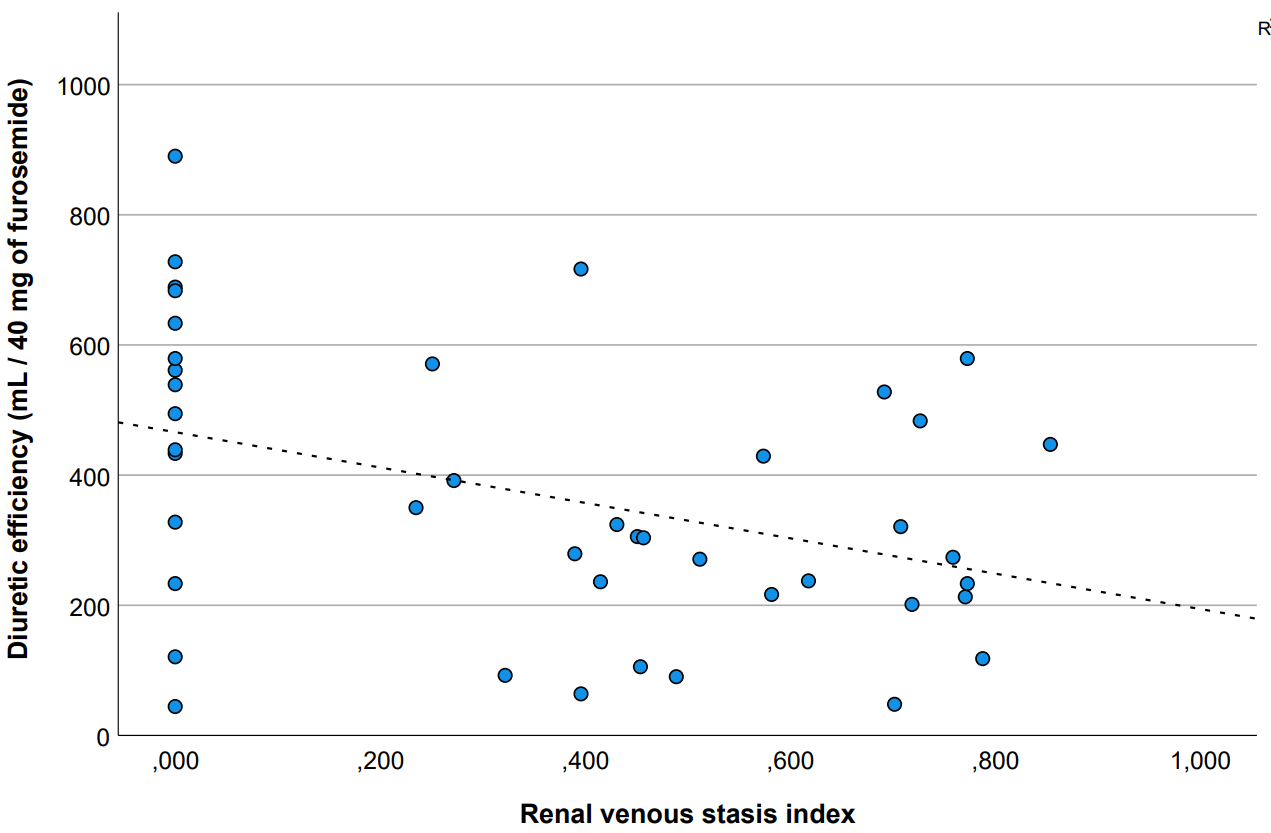


Legend: There is a moderate correlation between the renal venous stasis index and diuretic efficiency

(Spearman r=-,377 p=0.01)

Figure S3: Receiver operating characteristic plots illustrating the ability to discriminate high vs low diuretic efficiency based of the VExUS grading compared with A) individual components of the VExUS assessment and B) other clinical variables. (Low diuretic efficiency was defined as less than 325 mL of urine per 40 mg of furosemide equivalent.)

**
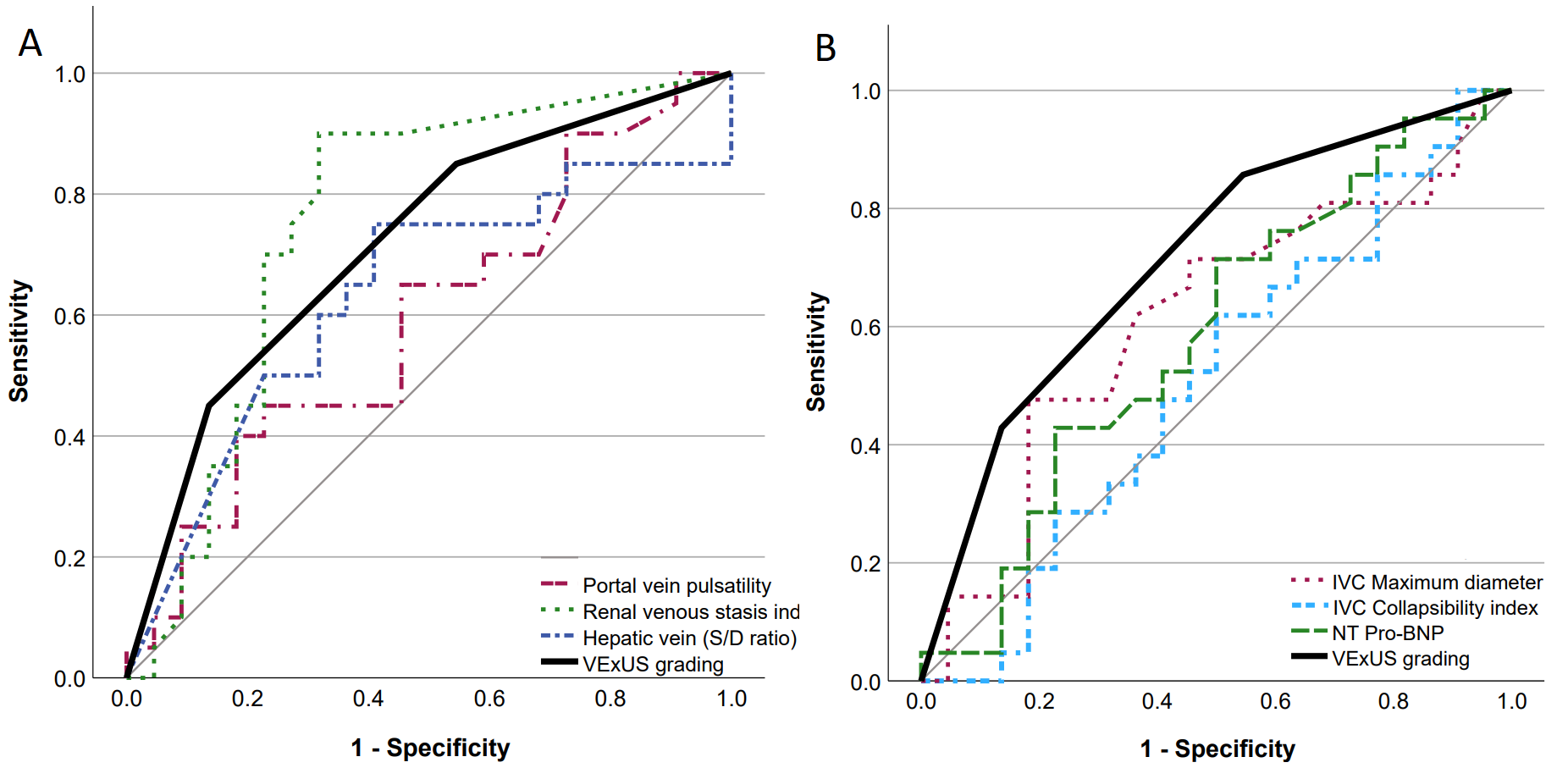
**

Figure S4: Percent change in ultrasound markers and central venous pressure from day 1 to day 3.


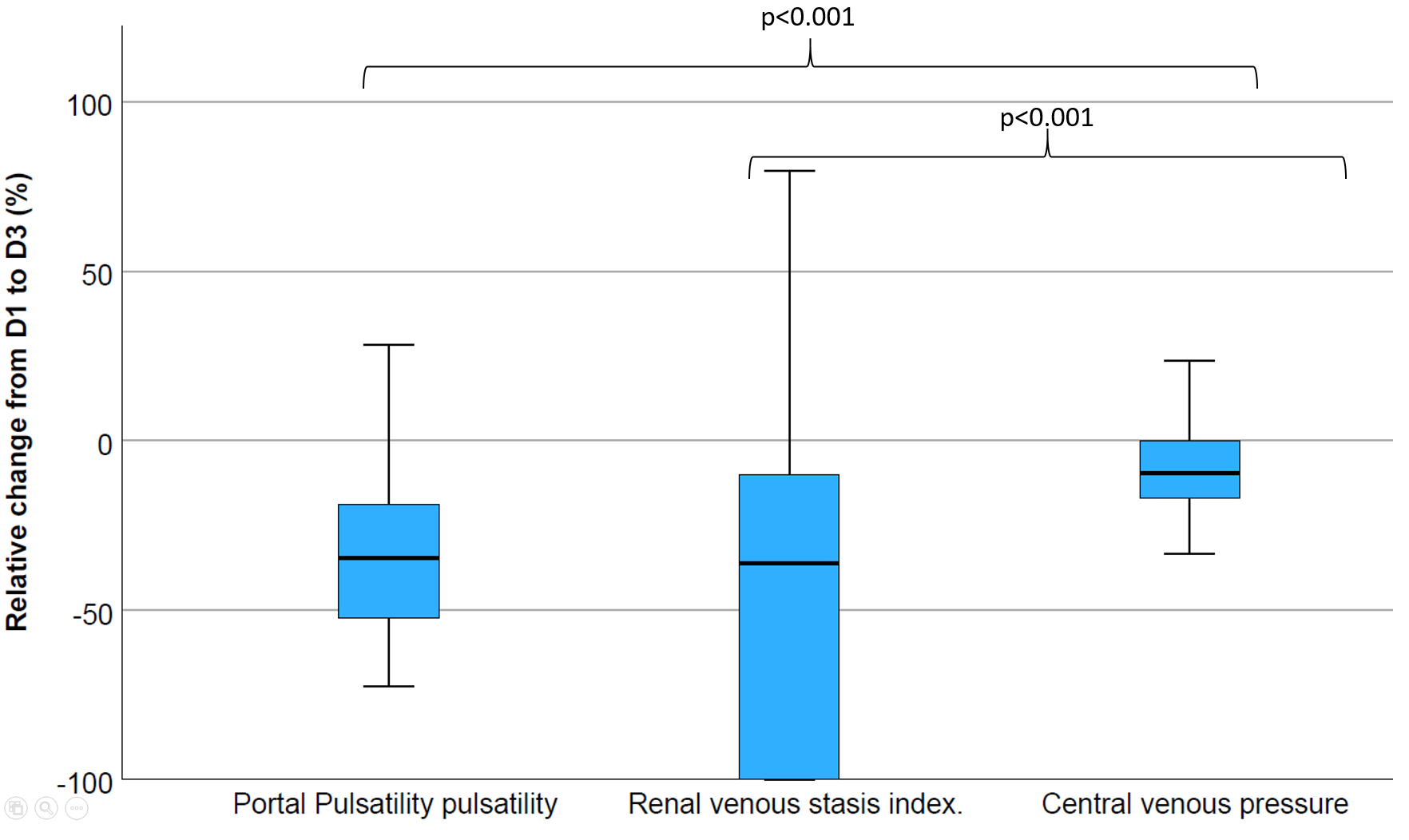

Supplement: Supplementary file 1 — Supplementary Material 1 [file 12882_2025_4060_MOESM1_ESM.docx]
